# Changes in Hepatitis E Virus Contamination during the Production of Liver Sausage from Naturally Contaminated Pig Liver and the Potential of Individual Production Parameters to Reduce Hepatitis E Virus Contamination in the Processing Chain

**DOI:** 10.3390/pathogens13040274

**Published:** 2024-03-23

**Authors:** Jan Bernd Hinrichs, Antonia Kreitlow, Lisa Siekmann, Madeleine Plötz, Nicole Kemper, Amir Abdulmawjood

**Affiliations:** 1 Institute of Food Quality and Food Safety, University of Veterinary Medicine Hannover, Foundation, 30173 Hannover, Germany; jan.bernd.hinrichs@tiho-hannover.de (J.B.H.); antonia.kreitlow@tiho-hannover.de (A.K.); lisa.siekmann@tiho-hannover.de (L.S.); madeleine.ploetz@tiho-hannover.de (M.P.); 2 Institute for Animal Hygiene, Animal Welfare and Farm Animal Behaviors, University of Veterinary Medicine Hannover, 30173 Hannover, Germany; nicole.kemper@tiho-hannover.de

**Keywords:** hepatitis E virus, reduction strategies, pork products, monitoring

## Abstract

In this study, changes in hepatitis E virus (HEV) contamination in the production of liver sausage from naturally contaminated pork liver were investigated. Furthermore, the potential effectiveness of individual production parameters in reducing viral loads was measured. When processing moderately contaminated liver (initial *Cq*-value 29), HEV RNA persisted in the finished sausages, even after heating for 90 min at 75 °C. A matrix-specific standard curve was created using a spiking experiment to accurately quantify HEV RNA in a particularly challenging matrix like liver sausage. Variations in product-specific production parameters, including mincing and heating times, showed some reduction in contamination levels, but even prolonged heating did not render all finished products HEV negative. The persistence of HEV contamination underscores the importance of ongoing monitoring in the pig population and raw materials to enhance food safety measures and reduce the likelihood of transmission through pork consumption. The detection of HEV RNA within all processing stages of pork liver in the production of liver sausage suggests that further research into the risk of infection posed by this detection and vigilance in managing HEV risks in the food chain, particularly in pork products, are required to protect public health.

## 1. Introduction

The hepatitis E virus (HEV), a member of the family *Hepeviridae*, is characterised by a non-enveloped or quasi-enveloped, single-stranded RNA genome of small size, approximately 7.2 kilobases in length [1]. The main route of infection is likely to be oral ingestion of the pathogen, with faecal–oral infection through water playing a major role in regions such as Southeast Asia and Africa [2]. In contrast, in North America, China and Europe, zoonotic transmission through the consumption of contaminated food of animal origin appears to be the main vector of infection [3]. Various studies have repeatedly shown a high prevalence of HEV antibodies in the human population of various European countries, with studies in Germany and France showing a prevalence of around 20% [4,5]. In Europe, the most common strain is HEV genotype 3 [6], which infects both humans and various other mammals [7], with domestic and wild boars considered to be important virus reservoirs [8]. Indeed, high antibody prevalences have also been repeatedly detected in the European pig population [9,10,11]. In recent years, tests on pigs at slaughterhouses have increasingly shown that a significant proportion of the animals and their organs, especially the liver, were contaminated with HEV [12,13]. However, as the disease hardly seems to lead to recognisable clinical symptoms in pigs [14], comprehensive screening in primary production is extremely difficult. Therefore, methods and tests for detecting HEV contaminations within the food chain and during the processing of raw materials are of great value [15].

As the virus mainly appears to infect hepatocytes and thus multiply primarily in the liver [16], foods containing pork liver in particular are suspected to be a source of HEV infections in consumers. HEV RNA has been repeatedly detected in various foods but especially in foods containing pork liver, which supports the assumption that products containing liver have a particularly high risk of contamination [17,18].

Also, several outbreaks of hepatitis E in Europe have been linked to foods containing pork liver, often because the food was not sufficiently heated [6,18,19]. This association may also explain the higher prevalence of anti-HEV-specific IgG in regions where products made from unheated liver are predominantly consumed, e.g., in the south of France or several regions of Italy [18]. 

The occurrence of HEV in food is probably largely due to the particularly high resistance of the virus against various environmental factors [20], as conventional antimicrobial methods in food processing have proven to be only marginally effective or even ineffective against the virus. For example, the virus was shown to have a high tolerance to nitrite salt [21], which is often used to cure sausages. In addition, HEV seems to be sensitive only to very low or high pH [22], and also the drying and smoking of meats seem not to significantly reduce the viral load [23]. More recent methods such as processing the material with UV light or high pressure show a certain effectiveness against the virus [24]. However, these place high technical demands on production and are mostly still undergoing theoretical testing [25]. Therefore, the best method for reducing HEV contamination seems to be heating. Several experiments have shown a significant reduction in viral load by appropriate heating steps [26], and some experiments have also investigated whether the remaining viral contamination is still infectious, either in cell culture [27] or in pig infection experiments [28].

However, less is known about the development of HEV contamination within industrial production processes. Based on the prevalence of HEV in the pig population, the processing of large quantities of liver will inevitably result in contamination, especially when large batches of pork liver are processed into liver sausage. This is further substantiated by the repeated detection of HEV RNA both in pork liver and in retail products made from it [17]. 

Unfortunately, many of the available results from prevalence studies in retail food products do not allow positive HEV RNA detections to be traced back within the production process. Additionally, quantitative analysis of the results is not usually performed, probably due to the fact that there are no standardised extraction and detection methods available for HEV RNA from different matrices [29]. 

The aim of this study was, therefore, to investigate how HEV contamination develops in industrial liver sausage production during the different production stages by using a previously established quantitative PCR assay [30]. Furthermore, the extent to which the processing steps commonly used in industrial production are suitable for reducing the prevalence of HEV RNA detection in the food chain and the points at which in-production screening may be useful were also the focus of the present study.

## 2. Materials and Methods

### 2.1. Production of Liver Sausage Based on an Industrial Recipe

**Figure 1:**
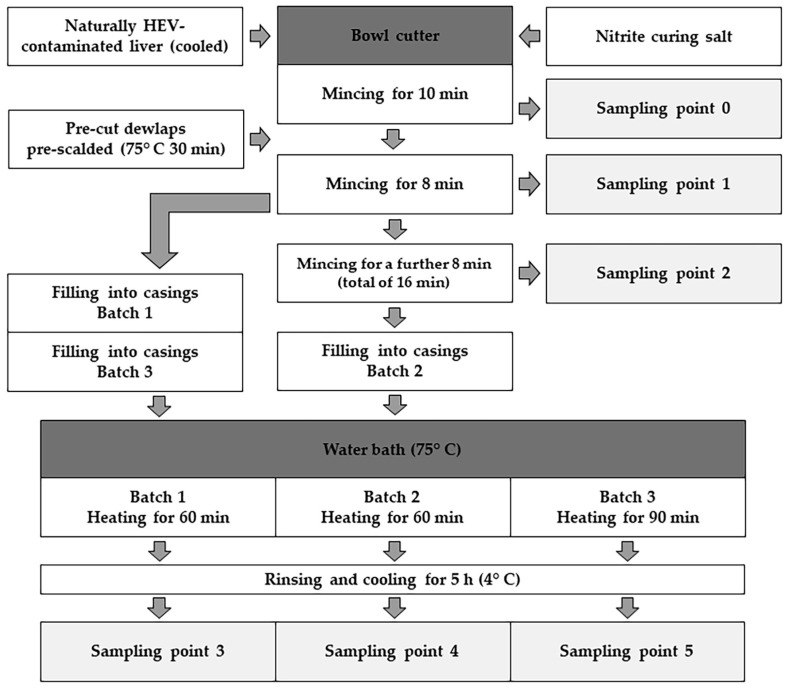
Production scheme and sampling points for the production of liver sausage from naturally contaminated pork liver.

To evaluate the changes in contamination within an industrial production process, the production scheme and proportions of the ingredients of an industrial recipe for liver sausage were used. The industrial recipe 4-001 for “Delikatessleberwurst” from the reference book *“Die Fabrikation feiner Fleisch- und Wurstwaren”* [31] was selected for this purpose, which includes a relatively long heating step in addition to steps for pre-cutting the individual ingredients. The quantities were adjusted to the weight of the processed liver so that a total quantity of around three kilogrammes of liver sausage was generally produced. For the production process, naturally contaminated pig liver sampled during the study by Hinrichs et al. [30] was used. The initial contamination in the used livers ranged from 38,880 GE/g to 8,624,000 GE/g [30]. The colour stabilisers, honey and spices specified in the recipe were omitted to avoid possible interferences from foreign DNA and potentially inhibiting substances on the molecular biological analysis during this study. Therefore, only liver, pre-cut defatted pork belly and nitrite curing salt were added to the bowl cutter. The pre-cut defatted pork bellies were pre-scalded at 75 °C for 30 min before being added to the cutter. The nitrite salt and chilled liver were first minced for 10 min until the homogenate began to bubble slightly. After adding the pre-scalded defatted pork bellies, the mass was minced for a further 8 min until it was completely homogenised. During this process, the temperature was kept below 25 °C in accordance with the recipe so as not to affect the binding properties of the sausage mixture. After mincing, the sausage mixture was filled into sterile 40/60 casings and heated at 75 °C for 60 min according to the production plan. The sausages were then removed, rinsed under cold water and analysed by PCR after five hours of storage at 4 °C (see Figure 1). 

### 2.2. Method for Extraction of HEV RNA

The extraction was performed on ice to avoid RNA losses in the sample. For this purpose, 50 g of material was first taken either (for samples within the production) from the centre of the initial production mixture in the bowl cutter or (for samples from finished sausages) from the sausage core, chopped with a scalpel and pre-mixed. A total weight of 0.25 g of sample material was then taken from this mixture and placed in a 2 mL reaction tube filled with zirconium beads and 250 µL of 1% phosphate-buffered saline (PBS). The sample was then homogenised using the Precellys-Evolution™ homogeniser (Bertin-Technologies SAS, Montigny-le-Bretonneux, France) for 5 cycles of 30 s each at a speed of 5 ms^−1^. The sample was then centrifuged at 13,000× *g* for 20 min at 5 °C. After piercing the floating fat layer with a pipette and dispensing the tip, 25 mg of the supernatant homogenate was taken from this tube and then added to a new tube containing 600 µL buffer RLT (Qiagen GmbH, Hilden, Germany) and homogenised again using a GK60 Precellys Lysing Kit™ (Bertin-Technologies) for 1 cycle of 20 s at a speed of 5 ms^−1^. Afterwards, the RNA was extracted and concentrated by ultrafiltration using the Qiagen RNeasy Kit™ (Qiagen GmbH) in accordance with the manufacturer’s recommendations and eluted in 50 µL RNase-free water. The eluate was immediately placed on ice and directly analysed using the quantitative RT-qPCR assay described by Hinrichs et al. [30].

### 2.3. Spiking Experiment for the Quantification of HEV RNA in Liver Sausage under Field Conditions

To quantify HEV contamination in the analysed samples as realistically as possible, a standard curve was created based on a spiking experiment. For this purpose, six portions of 0.25 g each of commercially bought liver sausage were prepared. The liver sausage was previously tested for the absence of HEV RNA using RT-qPCR. Homogenisation of the samples was carried out as described in Section 2.2. After the first homogenisation step, three 25 mg portions were taken from each of these six samples and transferred individually to a GK60 Precellys Lysing Kit™ (Bertin-Technologies SAS) containing 600 µL RLT buffer (Qiagen GmbH). Prior to the second homogenisation step, each of these 18 samples was spiked with 10 µL of a decimal dilution series of the target RNA, ranging from 1 × 10^6^ Genome equivalents (GE)/µL to 1 × 10^1^ GE/µL. The RNA used in this experiment was an oligonucleotide that was artificially produced and quantified by Eurofins Genomics Germany GmbH (Ebersberg, Germany) in accordance with the amplicon targeted by the PCR primer set in the ORF1 region of an HEV subtype 3c (accession number FJ705359 in the NCBI GenBank [https://www.ncbi.nlm.nih.gov/genbank/, accessed on 7 March 2023]).

After the second homogenisation step, the samples were further processed according to the Qiagen RNeasy Kit™ protocol; the isolated RNA was eluted in 50 µL RNase-free water and immediately placed on ice. With a complete isolation result for the spiked RNA, the concentrations in the resulting eluates were expected to be between 1 × 10^5^ GE/µL and 1 × 10^0^ GE/µL. All the spiked samples were tested in triplicate using the RT-qPCR described by Hinrichs et al. [30].

### 2.4. Investigating the Impact of Modified Production Parameters on Viral Loads

Three different batches were produced from the same HEV-contaminated production mixture to evaluate the potential of each production parameter to reduce the given HEV contamination. The mincing of the initial production mixture in a bowl cutter and the heating of the sausages were selected as deviating production parameters and potential critical points for the reduction of HEV contamination during industrial production. The comparison batch was produced according to the specified industrial recipe [Batch 1] (see Section 2.1) [31]. Therefore, after adding the defatted pork belly, the initial production mixture was minced for 8 min, and the filled sausage was heated at 75 °C for 1 h. For the second batch, 2/3 of the initial production mixture was removed from the bowl cutter after 8 min of mincing, and 1/3 of the mass was minced for a further 8 min to investigate the effects of improved cell disruption and potentially better heat exposure during the heating steps [Batch 2]. For the third batch, the production mixture was minced for 8 min according to the industrial recipe, but the filled sausage was heated at 75 °C for an extended time of 90 min [Batch 3]. All the batches were rinsed under cold water after heating and cooled at 4 °C for 5 h before testing.

### 2.5. Sampling Points during the Production Process

To track the contamination within this production process, samples were taken at three points during production and from each of the differently produced sausage batches (see Figure 1). The liver used in the production was analysed separately to determine the initial contamination level of the ingredients. Sample 1 was taken from the homogenate of the liver minced with nitrite curing salt for 10 min. After adding the defatted pork belly to the bowl cutter, sample 2 was taken from the ground mass after it had been minced for 8 min, so this sample represented the initial contamination of the ground mass. Sample 3 was taken from the production mixture after mincing for a further 8 min to evaluate any effects of prolonged mincing resulting in potentially greater homogenisation. The production mixture was then filled separately into casings in three different batches and heated at 75 °C for either 60 min or 90 min. After heating and cooling, the sausages were cut in half, and samples were taken from the centre of each batch of sausage (samples 3, 4 and 5). At each sampling time, three samples were taken and immediately analysed in triplicate.

Temperatures were also measured at four points during production and in each of the heated sausages to ensure that core temperatures were achieved and to assess whether certain changes in contamination were due to heat exposure.

## 3. Results

### 3.1. Spiking Experiment for the Quantification of HEV RNA in Liver Sausage under Field Conditions

To accurately quantify the results from the matrix, the liver sausage was spiked, and a matrix-specific standard curve was generated. As described in Section 2.3, 10 µL of each of the dilution levels 10^6^ to 10^1^ GE/µL was spiked so that a total of 10^7^ to 10^2^ GE was present in 25 mg of material. The corresponding results are shown in Table 1.

**Figure 2:**
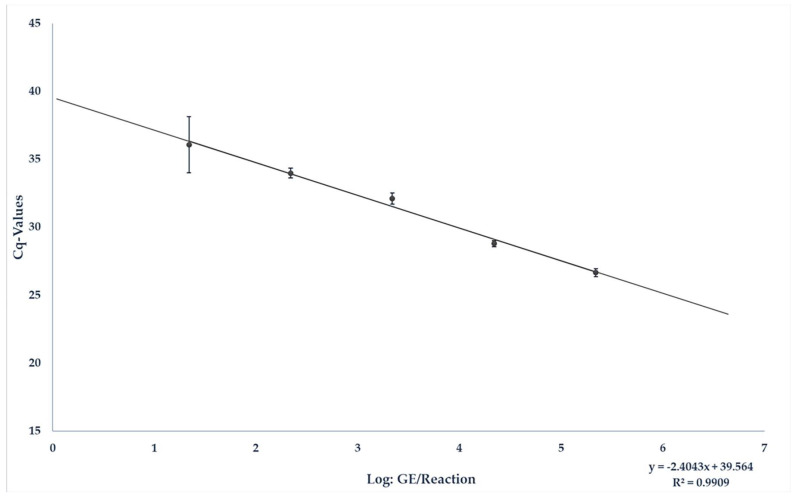
Standard curve from the results of measuring the analytical sensitivity of the JBH4-HEV RT-qPCR assay under field conditions by analysing liver sausage spiked with different dilution levels of target RNA.

The resulting standard curve could be described with the equation y = −2.403x + 39.564. The equation E = 10^(−1/−2.403)^ resulted in a PCR efficiency of 2.6056 and an R^2^ of 0.9909 (see Figure 2). The quantitative detection limit was set at 1 × 10^3^ GE/25 mg liver sausage. Positive results beneath the limit were approximated. The IAC (internal amplification control) was detected in every sample. An average *Cq*-value of 33.2 for the detection of the IAC was measured in the samples containing liver sausage, while an average *Cq*-value of 32.3 was measured in samples used as a positive control.

### 3.2. Development of HEV Contamination within the Production Process of Liver Sausage according to an Industrial Recipe

**Figure 3:**
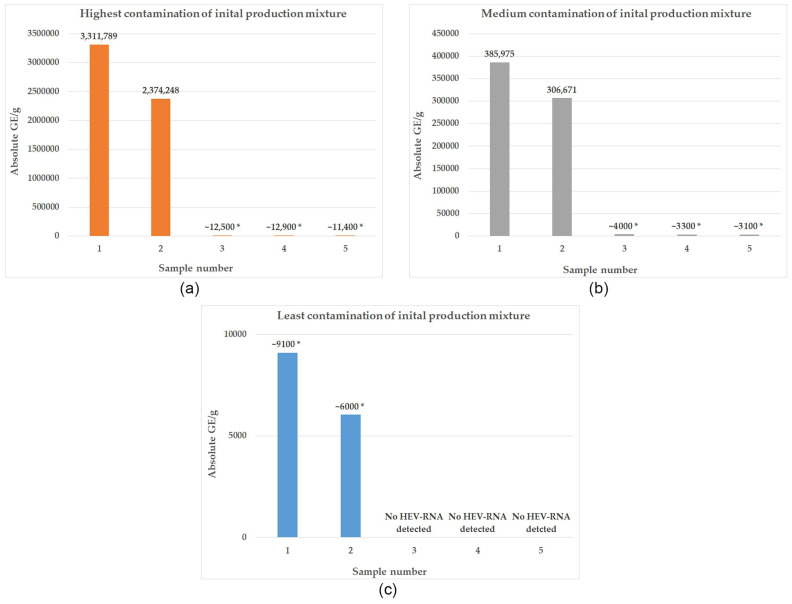
Changes in HEV contamination during three productions (**a**–**c**) of liver sausage from liver with varying degrees of natural HEV contamination; Sample No. 1: liver sausage production mixture (liver + nitrite curing salt + defatted pork belly), 8 min minced; Sample No. 2: liver sausage production mixture (liver + nitrite curing salt + defatted pork belly), 16 min minced; Sample No. 3: liver sausage—batch 1, 8 min minced, 60 min heated at 75 °C; Sample No. 4: liver sausage—batch 2, 16 min minced, 60 min heated at 75 °C; Sample No. 5: liver sausage—batch 3, 8 min minced, 90 min heated at 75 °C; * value estimated according to standard curve but beneath limit of quantification.

The naturally contaminated livers initially showed *Cq*-values between 24.7 and 32.4, which hardly changed after being minced with nitrite curing salt for 10 min, so that the results for sample 0 matched those for the naturally contaminated liver. The mean temperature of the minced livers in the bowl cutter before the addition of defatted pork bellies was 9.45 °C, and the temperature of the pre-scalded defatted pork bellies after heating and before being added to the bowl cutter was 48.23 °C. The initial *Cq*-values of the production mixture for production were found to be between 33.89 and 27.71 after 8 min of mincing. According to the standard curve determined for liver sausage, this corresponded to an initial viral load between ~9100 GE/g and 3,311,789 GE/g (see Figure 3). After filling, the liver sausages were heated in a water bath for 60 min at 75 °C according to the recipe. After this heating step, the sausages had an average temperature of 70.7 °C with a core temperature of 69.8 °C. HEV RNA was detected in 2/3 of the finished sausages produced under these conditions. The production with the highest initial contamination of the production mixture (3,311,789 GE/g) was positive, with the samples from the finished sausage having an average *Cq*-value of 33.557 (~12,500 GE/g), and the sausage produced from the production mixture with medium initial contamination (385,975 GE/g) had an average *Cq*-value of 34.75 (~4000 GE/g). No HEV RNA was detected in the sausage from the production mixture with the lowest initial load in nine replicates. The average reduction between the production mass and the finished sausages was 2.254 log.

### 3.3. Potential of Individual Production Parameters to Reduce Contamination

After 2/3 of the initial production mixture was removed from the bowl cutter, the temperature of the mass was 19.6 °C on average. After mincing the production mixture for a further 8 min, it had an average temperature of 17.53 °C. The samples taken from the longer-minced production mixture showed *Cq* values between 34.32 and 28.06, corresponding to ~6000 GE/g and 2,374,248 GE/g, respectively. HEV RNA was detected in 2/3 of the sausages produced from the production mixture that was minced for 16 min after heating for 60 min. An average *Cq*-value of 33.53 (12,800 GE/g) was detected in the finished sausage from the production with the highest initial contamination of the production mixture. An average *Cq*-value of 34.95 (~3300 GE/g) was detected in the sausage produced from the production mixture with medium initial contamination. No HEV RNA was detected in that batch of sausages produced from the lowest initial load in nine replicates. The average reduction was 2.278 log. In the batches of sausage that underwent additional heating for 30 min, HEV RNA was detected in 2/3 of the productions as well. The average temperature of that production batch was 72.8 °C after heating, with a core temperature of 72.4 °C. HEV RNA was detected in the production with the highest initial contamination of the production mixture, with an average *Cq*-value of 33.652 (~11,400 GE/g), and in the sausage produced from the production mixture with medium initial contamination, with an average *Cq*-value of 35.01 (~3100 GE/g). In the sausages produced from the lowest initial load and heated for 90 min, no HEV RNA was detected in nine replicates. The average reduction after heating for 90 min was 2.304 log.

## 4. Discussion

Alongside norovirus, hepatitis A virus and rotavirus, HEV is one of the most important food-associated viruses in Europe [18]. However, to date, much about the distribution of this virus and its development within the food chain is still unknown [32]. This is particularly problematic due to a rising number of reported cases in Europe [4]. A variety of foods are suspected to be vectors for the transmission of the virus [33], with pork products playing a major role in the spread of the virus [6].

The high prevalence of IgG antibodies against HEV in wild and domestic pig populations in Europe, which has been repeatedly observed in various studies, suggests that they appear to be an important virus reservoir [8]. In a randomised prevalence study of 186 pig farms in France, 31% of slaughter-aged pigs tested positive for HEV-specific IgG antibodies [9]; in a study in the Netherlands, the seroprevalence of pigs kept on organic farms was as high as 89% [34]. These results were also confirmed in more recent studies, such as by Boxman et al. (2022), in which an HEV-IgG prevalence of 67.6% was found in pigs at slaughterhouses in the Netherlands [11].

The resulting high risk of contamination of pig products poses a particular challenge for food safety, as HEV disease in pigs almost always takes a subclinical course and is, therefore, difficult to recognise in primary production, requiring the use of laboratory methods to detect infection and contamination [35]. 

The liver, which is the primary target organ of the virus, is particular susceptible to contamination with HEV [16]. Virus RNA has been repeatedly detected directly in pig liver, in studies of both wild boar and domestic pigs [12,18,36]. It was also found during the testing of liver samples at slaughterhouses and in retail [37], with up to 11% of domestic pig livers found to be positive for HEV RNA [11]. In many studies, HEV RNA has been detected in sausages containing liver, often at a higher prevalence than that in liver [18]. In addition to raw sausages containing liver, which are traditionally produced in Italy and southern France and have frequently tested positive for HEV RNA [38,39,40], HEV RNA has also been repeatedly detected with a high prevalence in partially heated liver sausages, which are more common in northern Europe [41,42,43]. The prevalence of HEV RNA in processed pork products often being even higher than that in pork liver itself could be due to the processing of large quantities of pig liver within industrial batches. At least, this significantly increases the risk of contaminated liver being introduced. This problem is exacerbated by the particularly high tenacity of HEV against the antimicrobial processes commonly used in the production of sausage and other products containing pork [21,22,23,24]. However, the extent to which the prevalence of HEV RNA detection can be reduced by industrial processing is still unclear.

The most effective method for inactivating and reducing HEV contamination, therefore, still appears to be heat treatment of the products [17]. Inactivation of the virus by treating infected liver at over 70 °C has been demonstrated both in animal experiments and in cell culture [27,28,44].

Schielke et al. [26] were able to detect a 99.9% reduction in virus contamination in a wild boar liver suspension containing HEV genotype 3 that was heated at 56 °C for 60 min and then analysed quantitatively by PCR. Johne et al. [27] found a 3.5-log reduction in virus contamination of cell cultures when heated between 65 °C and 75 °C, and no virus was detectable when heated at 70 °C for 2 min. Nonetheless, only few data are available for the reduction of HEV contamination within the specific production processes of foods susceptible to contamination. Imagawa et al. [45] and Feagins et al. [28] investigated HEV-spiked minced meat and naturally infected pork liver heated for different lengths of time and with different heating methods by testing infectivity in cell cultures and by oral inoculation of pigs. In both studies, the virus was found to be inactivated when heated at over 70 °C for 5 min. Baurnaud et al. [44] investigated the effect of different heating temperatures on pâté preparations spiked with infectious HEV by intravenous inoculation of pigs and found inactivation when heated at 71 °C for at least 20 min.

However, HEV RNA was still detectable after this treatment. Therefore, the products would have been considered HEV positive within testing. These results approximately correspond to the reduction observed in the present study within an industrial production process of liver sausage from HEV-contaminated pork liver. Nonetheless, the results also show that a reduction within the production process and the more difficult conditions caused by matrix-specific factors associated with it, such as higher fat content and different core temperatures, is more difficult to achieve than one under in vitro conditions. Even though there is still a need for further research, as this study can only provide insight into the virus development within the production process due to the relatively small number of samples analysed, the results show that even prolonged heating within a production process does not lead to products that appear HEV negative in molecular diagnostic testing. Even a relatively low-contaminated production mixture with an initial load of approx. 282,000 GE/g leads to an HEV-positive end product. This result is exacerbated by the fact that the HEV showed particularly high tenacity in in vitro storage tests and was still infectious after up to 28 days at room temperature [27], which could indicate a long-term risk of infection from contaminated liver in more complex matrices such as liver sausage, which is usually also stored refrigerated after production. It is very difficult to determine the specific risk of infection from this food, as no oral infectious dose has yet been established for humans. An oral dose of 10^6^ GE seems to reliably trigger virus excretion in pigs [14] and is, therefore, often assumed to be an infectious dose in humans as well [46]. This dose would be reached by the foods sampled here with a minimum consumption quantity of approx. 78 g. The quantification of viral loads in liver sausage remains difficult. This is because the extremely demanding matrix causes fluctuations in the reaction process, especially due to the inhibition of the detection reaction by its fat content [47], which was directly demonstrated in this study by a shift in *Cq*-values of the IAC in the spiking experiments themselves and by the comparison of the results reported in the literature for the quantification of liver with the PCR method used here [30]. This inhibition of individual samples in turn leads to relatively high fluctuations in the *Cq*-values, as visible in the results of the spiking experiment. However, since a high R^2^ value of almost 1 was achieved, the points were found to be well congruent, meaning that quantification can be reliably achieved [48]. Individual samples should be tested at least in triplicate to take any fluctuations occurring into account.

Furthermore, no conclusions can be drawn as to whether only free RNA or infectious virus RNA was detected [49]. Nonetheless, despite this uncertainty, large-scale foodborne infection risk assessment must rely on the detection of viral RNA [49] as functional and not-yet-standardised cell culture systems for HEV have only recently become available [32,50]. 

As shown in this study, HEV RNA can be detected in liver sausage even after an extensive heating step. To achieve a lower prevalence of HEV contamination in ready-to-eat foods in retail, it is, therefore, all the more important to monitor the spread of HEV in the pig population and the processed raw materials. Determining that the animals are HEV negative and carrying out a thorough screening of raw materials prior to and during production will most likely help to reduce HEV contamination and thus minimise infections [15,51].

## Figures and Tables

**Table 1:** Detection rate of target RNA in artificially contaminated liver sausage at different contamination levels.

GE/25 mg Sample	Positive/Tested	Average *Cq*-Value	Average Deviation
1 × 10^7^	9/9	25.86	1.29
1 × 10^6^	9/9	27.62	1.86
1 × 10^5^	9/9	31.61	0.9
1 × 10^4^	7/9	33.93	0.8
1 × 10^3^	7/9	36.1	4.47
1 × 10^2^	2/9	36.88	2.52

## Data Availability

Data are contained within the article.
